# National Early Warning Score 2 (NEWS2) on admission predicts severe disease and in-hospital mortality from Covid-19 – a prospective cohort study

**DOI:** 10.1186/s13049-020-00764-3

**Published:** 2020-07-13

**Authors:** Marius Myrstad, Håkon Ihle-Hansen, Anders Aune Tveita, Elizabeth Lyster Andersen, Ståle Nygård, Arnljot Tveit, Trygve Berge

**Affiliations:** 1 Department of Internal Medicine, Bærum Hospital Vestre Viken Hospital Trust, N-1346 Gjettum, Norway; 2 Department of Medical Research, Bærum Hospital Vestre Viken Hospital Trust, N-1346 Gjettum, Norway; 3 K.G. Jebsen Centre for B cell malignancies, Department of Immunology and Transfusion Medicine, Oslo University Hospital, N-0372 Oslo, Norway; 4 Department of Informatics, University of Oslo, N-0316 Oslo, Norway; 5 Oslo Center for Biostatistics and Epidemiology, Oslo University Hospital and University of Oslo, N-0317 Oslo, Norway; 6 Institute of Clinical Medicine, University of Oslo, N-0316 Oslo, Norway

**Keywords:** Covid-19, Coronavirus, qSOFA, NEWS2, Emergency department, Sensitivity, In-hospital mortality

## Abstract

**Background:**

There is a need for validated clinical risk scores to identify patients at risk of severe disease and to guide decision-making during the covid-19 pandemic. The National Early Warning Score 2 (NEWS2) is widely used in emergency medicine, but so far, no studies have evaluated its use in patients with covid-19. We aimed to study the performance of NEWS2 and compare commonly used clinical risk stratification tools at admission to predict risk of severe disease and in-hospital mortality in patients with covid-19.

**Methods:**

This was a prospective cohort study in a public non-university general hospital in the Oslo area, Norway, including a cohort of all 66 patients hospitalised with confirmed SARS-CoV-2 infection from the start of the pandemic; 13 who died during hospital stay and 53 who were discharged alive. Data were collected consecutively from March 9th to April 27th 2020. The main outcome was the ability of the NEWS2 score and other clinical risk scores at emergency department admission to predict severe disease and in-hospital mortality in covid-19 patients. We calculated sensitivity and specificity with 95% confidence intervals (CIs) for NEWS2 scores ≥5 and ≥ 6, quick Sequential Organ Failure Assessment (qSOFA) score ≥ 2, ≥2 Systemic Inflammatory Response Syndrome (SIRS) criteria, and CRB-65 score ≥ 2. Areas under the curve (AUCs) for the clinical risk scores were compared using DeLong’s test.

**Results:**

In total, 66 patients (mean age 67.9 years) were included. Of these, 23% developed severe disease. In-hospital mortality was 20%. Tachypnoea, hypoxemia and confusion at admission were more common in patients developing severe disease. A NEWS2 score ≥ 6 at admission predicted severe disease with 80.0% sensitivity and 84.3% specificity (Area Under the Curve (AUC) 0.822, 95% CI 0.690–0.953). NEWS2 was superior to qSOFA score ≥ 2 (AUC 0.624, 95% CI 0.446–0.810, *p* < 0.05) and other clinical risk scores for this purpose.

**Conclusion:**

NEWS2 score at hospital admission predicted severe disease and in-hospital mortality, and was superior to other widely used clinical risk scores in patients with covid-19.

## Background

The coronavirus disease 2019 (Covid-19) pandemic is straining health care systems worldwide. As of April 27th, 2020, more than 2,9 million confirmed cases and more than 200,000 deaths have been reported [1]. In Norway, the first case was reported on February 26th, 2020, and the cumulative incidence of reported cases has now reached 138 per 100,000 inhabitants, lower than the hardest affected countries in Europe [2].

The magnitude of the Covid-19 pandemic threatens the capacity and workflow of emergency departments (EDs) and intensive care units (ICU) in hospitals worldwide. Extra strain is imposed by limited knowledge of factors contributing to increased disease severity, and the significant proportion of hospitalised covid-19 patients that require respiratory support. Reports from the US and China, as well as European surveillance data, indicate that approximately 15–20% of hospitalised patients develop severe disease, defined as fatal outcome or a need for ICU treatment [2–4]. Traditional clinical risk stratification tools are widely used for triage and continuous assessment of patients with severe infectious disease, but few studies have evaluated the use of these tools in patients with SARS-CoV-2 infection. To overcome the challenges faced by the EDs during the pandemic, there is an urgent need for tools to efficiently identify patients at risk of severe disease, and thus help guide decision-making. The National Early Warning Score 2 (NEWS2), the quick Sequential Organ Failure Assessment (qSOFA), Systemic Inflammatory Response Syndrome (SIRS) criteria and CRB-65 are among the most commonly used clinical risk scores, but so far, there is a lack of evidence supporting their use in covid-19 patients [5]. We are not aware of any studies that have evaluated the ability of NEWS2 scoring at admission to predict outcome in patients hospitalised with SARS-CoV-2 infection.

## Methods

The aim of this study was to assess and compare the performance of NEWS2 and other commonly used clinical risk scores at ED admission to predict the development of severe disease and in-hospital mortality in patients with covid-19.

This is a prospective cohort study carried out at Bærum Hospital Vestre Viken Hospital Trust, a non-university hospital in the south-eastern part of Norway. The hospital serves approximately 185,000 inhabitants in two municipalities with more than 11,000 ED admissions per year. During the first weeks of the covid-19 outbreak in Norway, Bærum Hospital was among the hospitals in Norway with the highest rate of admission of covid-19 patients. The methods of the study and preliminary data have been previously published [6].

### Study population

Patients admitted to the ED and with confirmed covid-19 infection from March 9th 2020 were consecutively included in the study. Patients that were discharged or deceased up until April 27^th^ 2020 were included in the current analysis. Covid-19 was confirmed by qualitative detection of nucleic acid from SARS-CoV-2 in throat or nasal secretions by use of real-time polymerase chain reaction [7]. Patients with confirmed infection who were admitted for other reasons and did not have any symptoms or signs of covid-19 infection were excluded from the analysis based on clinical judgement.

### Measures and definitions

The first author registered all data used in this analysis by review of clinical scoring charts and patient records. Clinical scores were based on the first recorded vital signs after admission, documented on charts or in the records.

**Fig. 1:**
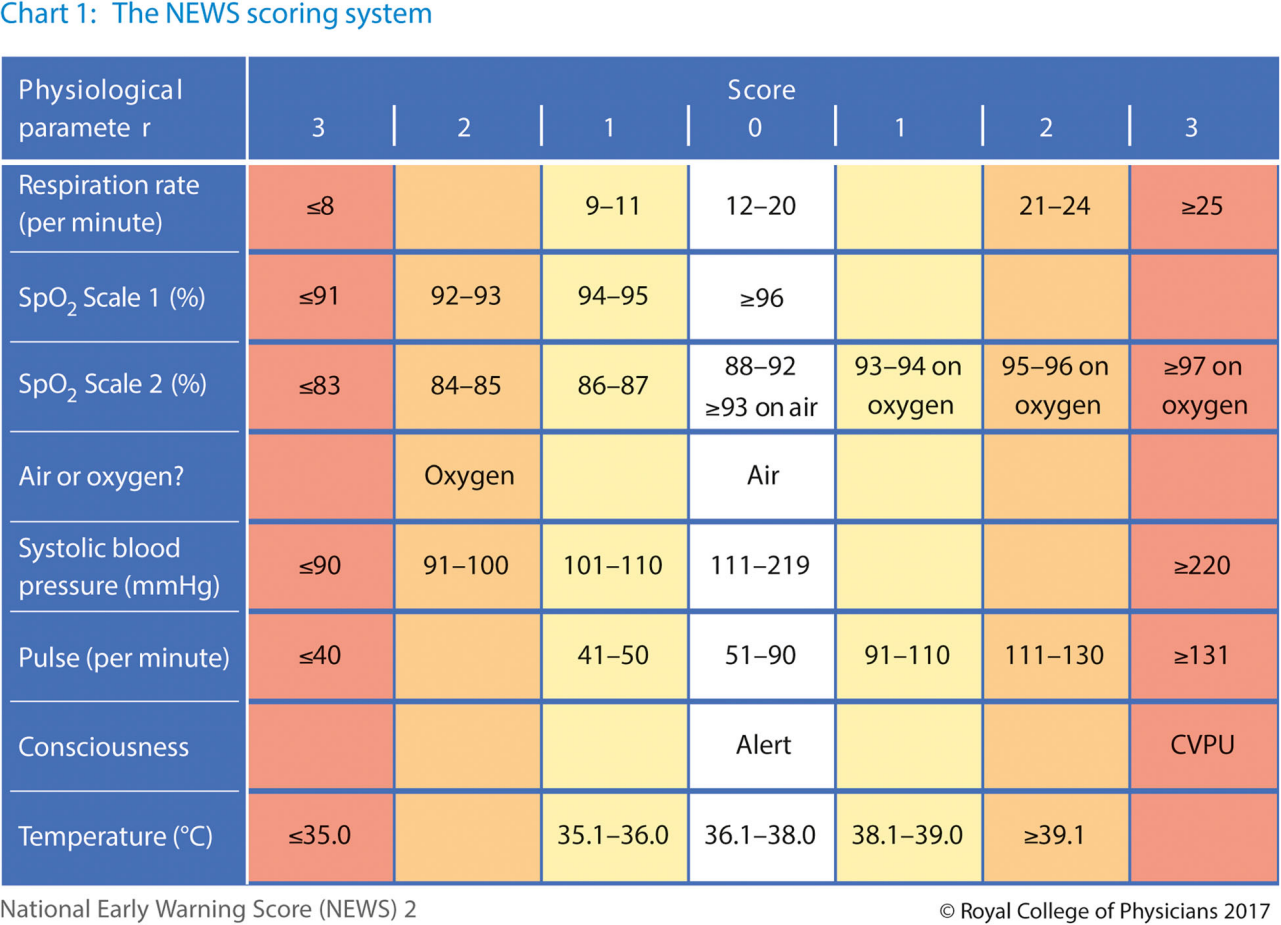
The NEWS2 scoring system. Reproduced from: Royal College of Physicians. *National Early Warning Score (NEWS) 2: Standardising the assessment of acute-illness severity in the NHS*. Updated report of a working party. London: RCP, 2017

*NEWS2* is a standardised clinical scoring system developed to improve detection of deterioration in acutely ill patients (Fig. 1) [8]. It is based on aggregate scoring of six physiological parameters; respiratory rate, oxygen saturation, systolic blood pressure, pulse rate, level of consciousness or new confusion, and body temperature. In addition, two points are added for patients requiring supplementary oxygen treatment. A NEWS2 score of 5 or 6 is considered a key threshold that may indicate clinical deterioration and should prompt urgent response by a clinician or a team with competence in assessment and treatment of acutely ill patients [8]. The NEWS2 scoring chart is utilised as part of routine patient care practice at our hospital.

*qSOFA* has been recommended as the tool of choice to assess organ dysfunction in patients with suspected sepsis [9]. Three clinical variables: altered mental status, systolic blood pressure ≤ 100 mmHg, and respiratory rate of ≥22/min; are scored with one point each. A qSOFA sum score ≥ 2 should prompt clinicians to investigate for organ dysfunction, initiate or escalate therapy, and to consider increased monitoring or referral to an ICU.

*SIRS* was defined as an evident infection with the presence of two or more of the criteria temperature > 38 °C or < 36 °C, heart rate > 90, respiratory rate > 20 or PaCO_2_ < 32 mmHg, and white blood cells > 12,000/mm^3^ or < 4000/mm^3^ at admission [10].

*CRB-65* is a clinical score developed for risk stratification of patients with community-acquired pneumonia. One point each is given for the clinical variables new confusion, respiratory rate ≥ 30, and systolic blood pressure < 90 mmHg or diastolic blood pressure ≤ 60 mmHg. In addition, age ≥ 65 years is scored with one point. A score of two or more indicates a need for hospitalisation and in-patient management [11].

We defined *severe disease* as a composite measure of death during hospitalization or ICU treatment for any reason during the hospital stay. *In-hospital mortality* was defined as death during hospital stay for any reason, related or unrelated to the covid-19 infection.

We used the Charlson Charlson Comorbidity Index (CCI) and the Clinical Frailty Scale (CFS) to characterise the premorbid status of the study population. CCI assesses chronic comorbidities such as heart failure, chronic kidney disease, chronic obstructive pulmonary disease (COPD) and malignancy, and predicts mortality in hospitalised patients [12]. CCI was scored based on comorbidities documented in patient records. CFS is a tool that is used to rapidly summarise the overall level of fitness or frailty based on the functional capacity of the patient 14 days prior to the onset of acute illness [13]. CFS was scored based on information about the patients functional status documented in hospital records. Body mass index (BMI) was calculated based on patient height and weight registered during the hospital stay. Smoking habits were self-reported at admission.

### Statistical methods

We used the registry tool EpiData entry client version 4.4.3.1 (The EpiData Association, Odense, Denmark). Continuous variables are presented as the mean ± standard deviation and categorical variables as the number (%). We used Student’s t test for means of continuous variables and Pearson’s Chi-square test of independence for categorical variables to compare characteristics of patient subgroups. Data was missing on smoking for six patients and BMI for 17 patients. To assess the ability of clinical tools to predict severe disease and in-hospital mortality, we calculated sensitivity, specificity, and positive and negative predictive values with 95% confidence intervals (CIs) using MEDCALC statistical software (http://www.medcalc.org). We used cut-off values that are recommended and commonly used; NEWS2 scores ≥5 and ≥ 6, qSOFA score ≥ 2, ≥2 SIRS criteria, and CRB-65 score ≥ 2. Areas under the curve (AUCs) for the clinical risk scores were compared using DeLong’s test implemented in the R package pROC (R version 3.6.1, The R Foundation) [14]. All other statistical analyses were conducted using SPSS version 25.0 (IBM, Armonk, NY, USA).

### Patient and public involvement

Patient or public involvement in the design, execution or dissemination of results of the present study was not considered feasible or relevant.

### Ethical considerations

The study was approved by the Vestre Viken Hospital Trust institutional review. Since only routine clinical data were collected from the electronic health records, the requirement for informed consent was waived. A letter with information about the study was sent by post to all patients, allowing the patient to withdraw their data. The study complies with the Declaration of Helsinki. Due to the proximity to real time, the interests of protection of privacy and the uncertainty implicit in a limited dataset, we refrained from detailed characterization of deceased patients, and analysed patients with severe disease and deceased patients as one category.

## Results

During 50 days, from March 9th until April 27th, 75 patients were included in the study. Seven patients who were still under treatment, and two patients with positive SARS-CoV-2 test, but without any symptoms or signs of clinically relevant covid-19 infection, were excluded, leaving 66 eligible patients for the current analysis.

The mean age was 67.9 (median 71.5, range 30–95) years, 38 out of 66 patients (58%) were men. A total of 15 patients (23%) were aged ≥80 years, 19 (29%) had a CFS score ≥ 5, indicating frailty. Mean CCI was 3.5, and the most common co-morbidities were hypertension and cardiac conditions.

Fifteen patients (23%) had severe disease, and in-hospital mortality was 20% (13 out of 66 patients). Seven patients were treated in the ICU, five out of seven patients were transferred to the ICU within 24 h after hospital admission. Median number of days from admission to transfer to ICU or death was five (mean 5.5 days). Table 1 shows patient characteristics by disease severity. Patients with severe disease were older, had a higher mean CCI, and were more likely to be men and have COPD than patients with less severe disease. Patients with severe disease more often presented with newly onset confusion and dyspnoea at admission.

**Table 1:** Characteristics and symptoms at admission in hospitalised patients with covid-19 admitted in the period from March 9th to April 27th 2020 by disease severity, *n* = 66

	Severe disease (***n*** = 15)	Less severe disease (***n*** = 51)	***p***-value
	Mean (SD)	Mean (SD)	
Age (years)	75.8 (13.6)	65.6 (16.4)	< 0.05
Clinical Frailty Scale score^1^	3.7 (2.4)	3.0 (2.0)	0.31
Charlson Comorbidity Index	5.3 (3.9)	2.9 (2.5)	< 0.05
Body mass index (kilograms/m^2^)^2^	23.4 (4.2)	26.3 (4.2)	0.09
Days of symptoms prior to admission	6.9 (3.8)	8.7 (5.0)	0.15
	**n (%)**	**n (%)**	
Male gender	12 (80)	26 (51)	< 0.05
Admitted from nursing home	2 (13)	2 (4)	0.31
Current or previous smoking^3^	4 (27)	14 (33)	0.67
*Clinical Frailty Scale score*			0.10
1–2 (fit)	7 (47)	24 (47)	
3–4 (managing well or vulnerable)	1 (7)	15 (29)	
≥ 5 (moderately or severely frail)	7 (47)	12 (24)	
*Charlson Comorbidity Index*			0.10
0–2	3 (20)	25 (49)	
3–5	7 (47)	18 (35)	
> 5	5 (33)	8 (16)	
*Co-morbidities*			
Hypertension	4 (27)	15 (29)	0.84
Diabetes mellitus	3 (20)	5 (10)	0.36
Cardiac conditions	3 (20)	8 (16)	0.69
Chronic kidney disease	2 (13)	5 (10)	0.70
Malignancy	3 (20)	6 (12)	0.41
Asthma	0 (0)	7 (14)	0.13
COPD^4^	4 (27)	1 (2)	< 0.05
Others	2 (13)	9 (18)	0.69
*Symptoms*			
Cough	8 (53)	32 (63)	0.51
Dyspnoea	13 (87)	30 (59)	< 0.05
Fever	10 (67)	36 (71)	0.77
Reduced general condition	14 (93)	39 (76)	0.15
New confusion	5 (33)	6 (12)	< 0.05
Gastrointestinal	5 (33)	9 (18)	0.19
Others	1 (7)	21 (41)	< 0.05

^1^ Based on functional status 14 days prior to acute illness. ^2^ Missing data for 17 patients (6 with severe illness and 11 with less severe illness). ^3^ Missing data for 8 patients with less severe illness. ^4^ Chronic obstructive pulmonary disease

**Fig. 2:**
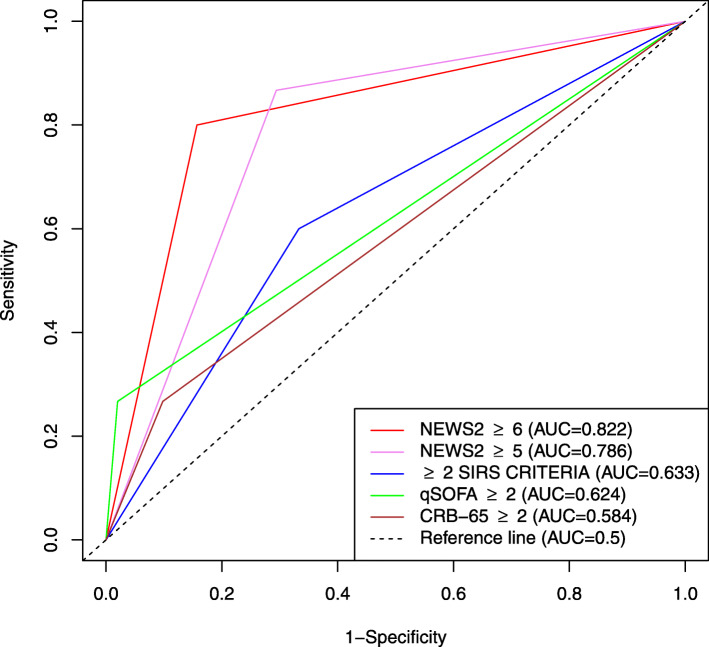
Receiver Operator Characteristic curves illustrating the ability of NEWS2 scores ≥5 and ≥ 6, and qSOFA score ≥ 2, CRB-65 score ≥ 2 and ≥ 2 SIRS criteria at emergency department admission to predict severe disease from covid-19, *n* = 66

In total, 28 patients (42%) had a NEWS2 score ≥ 5 and 20 (30%) had a NEWS2 score ≥ 6 at admission. Only five patients (8%) presented with a qSOFA score ≥ 2. Table 2 shows vital signs and clinical risk scores at admission by disease severity. Table 3 and Fig. 2 shows the performance of the studied clinical risk scores to predict severe disease. The highest AUC was found for a NEWS2 score ≥ 6. NEWS2 ≥ 6 also predicted in-hospital mortality with the highest AUC (0.790, 95% CI 0.643–0.937), with 76.9% (95% CI 46.2–94.7) sensitivity and 80.1% (95% CI 68.0–90.6) specificity (Additional file 1). NEWS2 was superior to qSOFA and other clinical risk scores to predict severe disease (Additional file 2).

**Table 2:** Vital signs and clinical risk scores at emergency department admission in patients with covid-19 infection in the period from March 9th to April 27th by disease severity, n = 66

	Severe disease (n = 15)	Less severe disease (n = 51)	p-value
	Mean (SD)	Mean (SD)	
*Vital signs*			
Body temperature (°C)	38.0 (0.8)	38.0 (0.7)	0.99
Respiratory rate (breaths/min)	26.3 (6.3)	20.1 (3.3)	< 0.05
Oxygen saturation (%)	92.2 (3.0)	95.7 (2.7)	< 0.05
Heart rate (beats/min)	89.1 (14.7)	80.3 (18.2)	0.07
Systolic blood pressure (mmHg)	127.7 (21.5)	132.1 (19.8)	0.49
NEWS2^1^ score	6.9 (3.2)	3.4 (2.3)	< 0.05
	**n (%)**	**n (%)**	
*Clinical risk scores*			
NEWS2^1^ score ≥ 5	13 (87)	15 (29)	< 0.05
NEWS2^1^ score ≥ 6	12 (80)	8 (16)	< 0.05
qSOFA^2^ score ≥ 2	4 (27)	1 (2)	< 0.05
≥ 2 SIRS^3^ criteria	9 (60)	17 (33)	0.06
CRB-65 score ≥ 2	4 (27)	5 (10)	0.09
*Selected subscores*			
Oxygen saturation ≤ 93%	9 (60)	9 (18)	< 0.05
Respiratory rate ≥ 22/min	11 (73)	15 (29)	< 0.05
Systolic blood pressure ≤ 100 mmHg	1 (7)	1 (2)	0.35
Heart rate > 90 beats/min	5 (33)	10 (20)	0.27
Acute confusion	5 (33)	7 (14)	0.08
Glasgow Coma Scale score ≤ 14	4 (27)	3 (6)	< 0.05

^1^ National Early Warning Score. ^2^ Quick Sequential Organ Failure Assessment. ^3^ Systemic Inflammatory Response Syndrome

**Table 3:** Performance of clinical risk scores at emergency department admission to predict severe disease from covid-19, n = 66

	Sensitivity % (95% CI)	Specificity % (95% CI)	PPV % (95% CI)	NPV % (95% CI)	AUC (95% CI)
**NEWS2 ≥ 5**	86.7 (59.5–98.3)	70.6 (56.2–82.5)	46.4 (35.2–58.1)	94.7 (83.0–98.5)	0.786 (0.659–0.913)
**NEWS2 ≥ 6**	80.0 (52.9–95.7)	84.3 (71.4–92.9)	60.0 (43.1–74.8)	93.5 (83.8–97.5)	0.822 (0.690–0.953)
**qSOFA ≥ 2**	26.7 (7.8–55.1)	98.0 (89.6–100.0)	80.0 (32.6–97.1)	82.0 (77.0–86.1)	0.624 (0.446–0.810)
**≥2 SIRS criteria**	60.0 (32.3–83.7)	66.7 (52.1–79.2)	34.6 (23.1–48.3)	85.0 (74.8–91.6)	0.633 (0.470–0.796)
**CRB-65 ≥ 2**	26.7 (7.8–55.1)	90.2 (78.6–96.7)	44.4 (19.7–72.3)	80.7 (75.3–85.2)	0.584 (0.410–0.759)

CI, Confidence interval; PPV, Positive Predictive Value; NPV, Negative Predictive Value; AUC, Area under the Curve; NEWS, National Early Warning Score; qSOFA, Quick Sequential Organ Failure Assessment; SIRS, Systemic Inflammatory Response Syndrome

## Discussion

The principal finding of the current study is that a NEWS2 score at hospital admission appears superior to qSOFA and other widely used clinical risk scores in prediction of severe disease and in-hospital mortality from covid-19. The high proportion with severe disease among hospitalised patients and the in-hospital mortality of 20% is in line with recently published reports from other settings [2–4, 15].

In the current pandemic situation, early identification of patients at risk of severe disease, and decision of in-hospital level of care, is crucial. NEWS2 is widely used in clinical practice, but its use in the context of covid-19 has so far not been evaluated. A Chinese group suggested an adapted version of the NEWS2 score with the addition of age > 65 years (3 points), reflecting evidence that increased age is associated with poor prognosis [16]. Respiratory failure is a hallmark in covid-19 patients, often without accompanying circulatory failure [3]. Several case reports have described the presence of hypoxemia without evident symptoms of respiratory distress, so-called ‘silent hypoxemia’ [17, 18]. An advantage of NEWS2 compared to the other studied tools is that both hypoxemia and supportive oxygen treatment are included as scoring parameters. In spite of a lack of evidence, the UK Royal College of Physicians recommends the use of the NEWS2 in the management of covid-19, but stresses the fact that any increase in oxygen requirements should trigger further evaluation [19]. Of note, increased oxygen requirement may not be reflected in the NEWS2 score, in which oxygen supplementation is only a binary variable (yes/no).

Larger studies are needed to confirm the results of our study, and to investigate the optimal cut-off value for clinical use. Only 2 of 15 patients who developed severe disease had a NEWS2 score lower than 5 at admission. Both these patients were aged > 75 years and died during the hospital stay, without ICU treatment. Elderly patients present with less typical and less pronounced symptoms than younger, and clinical risk scores should be used with caution in these patients. While the clinical scores in our study were based on the first recorded signs after ED admission, a recently published case series of 17 patients aged ≥80 years suggest that the variability in NEWS2 scores rather than a single observation at admission could predict poor outcome [20].

Our study indicates that qSOFA, CRB-65 and SIRS criteria at admission are less able to predict severe disease in patients with covid-19. Thus, these clinical risk scores should be applied with great care in the evaluation of covid-19 patient, and with increased awareness of other clinical signs, in particular signs of respiratory distress and hypoxemia.

### Strengths and limitations

The current study has limitations, the small study size being the most prominent. Furthermore, a single time point clinical evaluation directly after admission allows only a snapshot of the patient’s disease course. Patients with severe disease might have been more severely ill already at admission, for instance due to patient delay. However, the mean number of days with symptoms before admission did not differ between patients with severe and less severe disease. Our results may be considered preliminary, providing a strong case for further studies evaluating the ability of NEWS2 to predict poor outcomes in hospitalised patients with covid-19. Studies evaluating NEWS2 at admission and follow-up should be performed in larger prospective cohorts. Furthermore, data on height, weight, comorbidity and frailty were collected retrospectively based on information from hospital records. Missing data for BMI limits the ability of the study to evaluate the role of overweight to predict severe disease.

Main strengths of the study are that we have consecutively included all covid-19 patients admitted from the start of the outbreak, and that our data on vital signs at hospital admission, including all components of the NEWS2 score, are complete.

## Conclusions

One out of four patients hospitalised with covid-19 had severe disease, and in-hospital mortality was 20%. NEWS2 score at emergency department admission predicted severe disease and in-hospital mortality and was superior to qSOFA and other clinical risk scores for this purpose. A NEWS2 score ≥ 6 predicted severe disease with 80.0% sensitivity and 84.3% specificity. The use of clinical scoring systems to predict severe disease and mortality in patients with covid-19 should be investigated further in larger prospective studies.

## Supplementary information

**Additional file 1 Table S1.** Performance of clinical risk scores at emergency department admission to predict in hospital mortality in patients with covid-19, *n* = 66.

**Additional file 2 Table S2.** Comparison of the areas under receiver operating characteristic curves for the ability of clinical risk scores to predict severe disease and in-hospital mortality from covid-19.

## Data Availability

The datasets generated and/or analysed during the current study are not publicly available, but are available from the corresponding author on reasonable request.
