# Supplementary material for: National Early Warning Score 2 (NEWS2) on admission predicts severe disease and in-hospital mortality from Covid-19 – a prospective cohort study
Source: Scand J Trauma Resusc Emerg Med. 2020 Jul 13;28:66. doi: 10.1186/s13049-020-00764-3 (PMC7356106; doi:10.1186/s13049-020-00764-3)
Supplement: Supplementary file 1 — Additional file 1 Table S1. Performance of clinical risk scores at emergency department admission to predict in hospital mortality in patients with covid-19, n = 66. [file 13049_2020_764_MOESM1_ESM.docx]

**Supplementary table 1.** Performance of clinical risk scores at emergency department admission to predict in hospital mortality in patients with covid-19, n=66.

|  | **Sensitivity %**  **(95% CI)** | **Specificity %**  **(95% CI)** | **PPV %**  **(95% CI)** | **NPV %**  **(95% CI)** | **AUC**  **(95% CI)** |
| --- | --- | --- | --- | --- | --- |
|  |  |  |  |  |  |
|  |  |  |  |  |  |
| **NEWS2 ≥5** | 84.6  (54.6-98.1) | 67.9  (53.7-80.1) | 39.3  (29.1-50.5) | 94.7  (83.2-98.5) | 0.763  (0.624-0.902) |
| **NEWS2 ≥6** | 76.9  (46.2-94.7) | 80.1  (68.0-90.6) | 50.0  (34.7-65.3) | 93.5  (84.0-97.5) | 0.790  (0.643-0.937) |
| **qSOFA ≥2** | 30.8  (9.1-61.4) | 98.1  (89.9-100.0) | 80.0  (32.8-97.1) | 85.3  (80.1-89.3) | 0.644  (0.455-0.834) |
| **≥2 SIRS criteria** | 53.9  (25.1-80.8) | 64.2  (49.8-76.9) | 26.9  (16.6-40.6) | 85.0  (75.3-91.3) | 0.590  (0.414-0.766) |
| **CRB-65 ≥2** | 30.8  (9.1-61.4) | 90.6  (79.3-96.9) | 44.4  (20.0-72.0) | 84.2  (78.6-88.6) | 0.607  (0.421-0.792) |

CI, Confidence interval; PPV, Positive Predictive Value; NPV, Negative Predictive Value; AUC, Area under the Curve; NEWS, National Early Warning Score; qSOFA, Quick Sequential Organ Failure Assessment; SIRS, Systemic Inflammatory Response Syndrome.
