# Supplementary material for: National Early Warning Score 2 (NEWS2) on admission predicts severe disease and in-hospital mortality from Covid-19 – a prospective cohort study
Source: Scand J Trauma Resusc Emerg Med. 2020 Jul 13;28:66. doi: 10.1186/s13049-020-00764-3 (PMC7356106; doi:10.1186/s13049-020-00764-3)
Supplement: Supplementary file 2 — Additional file 2 Table S2. Comparison of the areas under receiver operating characteristic curves for the ability of clinical risk scores to predict severe disease and in-hospital mortality from covid-19. [file 13049_2020_764_MOESM2_ESM.docx]

**Supplementary table 2.** Comparison of the areas under receiver operating characteristic curves for the ability of clinical risk scores to predict severe disease and in-hospital mortality from covid-19.

| Comparison of Areas Under the Curve | p-value |
| --- | --- |
|  |  |
| *Severe disease* |  |
| NEWS2 ≥6 vs. qSOFA≥2 | 0.005 |
| NEWS2 ≥5 vs. qSOFA≥2 | 0.03 |
| NEWS2 ≥6 vs. ≥2 SIRS criteria | 0.04 |
| NEWS2 ≥5 vs. ≥2 SIRS criteria | 0.07 |
| NEWS2 ≥6 vs. CRB-65 ≥2 | 0.001 |
| NEWS2 ≥5 vs. CRB-65 ≥2 | 0.007 |
|  |  |
| *In-hospital mortality* |  |
| NEWS2 ≥6 vs. qSOFA≥2 | 0.06 |
| NEWS2 ≥5 vs. qSOFA≥2 | 0.13 |
| NEWS2 ≥6 vs. ≥2 SIRS criteria | 0.06 |
| NEWS2 ≥5 vs. ≥2 SIRS criteria | 0.07 |
| NEWS2 ≥6 vs. CRB-65 ≥2 | 0.02 |
| NEWS2 ≥5 vs. CRB-65 ≥2 | 0.05 |

NEWS, National Early Warning Score; qSOFA, Quick Sequential Organ Failure Assessment; SIRS, Systemic Inflammatory Response Syndrome.
